# Involvement of Allosteric Effect and K_Ca_ Channels in Crosstalk between β_2_-Adrenergic and Muscarinic M_2_ Receptors in Airway Smooth Muscle

**DOI:** 10.3390/ijms19071999

**Published:** 2018-07-09

**Authors:** Hiroaki Kume, Osamu Nishiyama, Takaaki Isoya, Yuji Higashimoto, Yuji Tohda, Yukihiro Noda

**Affiliations:** 1 Department of Respiratory Medicine and Allergology, Faculty of Medicine, Kindai University, 377-2 Ohnohigashi, Osakasayama 589-8511, Japan; nishi-o@med.kindai.ac.jp (O.N.); solution.management27@gmail.com (T.I.); yhigashi@med.kindai.ac.jp (Y.H.); tohda@med.kindai.ac.jp (Y.T.); 2 Division of Clinical Sciences and Neuropsychopharmacology, Graduate School of Pharmacy, Meijo University, 150 Yagotoyama, Tempaku-ku, Nagoya 468-8503, Japan; y-noda@med.nagoya-u.ac.jp

**Keywords:** synergistic effects, G protein, large-conductance Ca^2+^-activated K^+^ channels, L-type voltage-dependent Ca^2+^ channels, β_2_-adrenoceptor agonists, muscarinic receptor antagonists, asthma, COPD

## Abstract

To advance the development of bronchodilators for asthma and chronic obstructive pulmonary disease (COPD), this study was designed to investigate the mechanism of functional antagonism between β_2_-adrenergic and muscarinic M_2_ receptors, focusing on allosteric effects and G proteins/ion channels coupling. Muscarinic receptor antagonists (tiotropium, glycopyrronium, atropine) synergistically enhanced the relaxant effects of β_2_-adrenergic receptor agonists (procaterol, salbutamol, formoterol) in guinea pig trachealis. This crosstalk was inhibited by iberitoxin, a large-conductance Ca^2+^-activated K^+^ (K_Ca_) channel inhibitor, whereas it was increased by verapamil, a L-type voltage-dependent Ca^2+^ (VDC) channel inhibitor; additionally, it was enhanced after tissues were incubated with pertussis or cholera toxin. This synergism converges in the G proteins (G_i_, G_s_)/K_Ca_ channel/VDC channel linkages. Muscarinic receptor antagonists competitively suppressed, whereas, β_2_-adrenergic receptor agonists noncompetitively suppressed muscarinic contraction. In concentration-inhibition curves for β_2_-adrenergic receptor agonists with muscarinic receptor antagonists, EC_50_ was markedly decreased, and maximal inhibition was markedly increased. Hence, muscarinic receptor antagonists do not bind to allosteric sites on muscarinic receptors. β_2_-Adrenergic receptor agonists bind to allosteric sites on these receptors; their intrinsic efficacy is attenuated by allosteric modulation (partial agonism). Muscarinic receptor antagonists enhance affinity and efficacy of β_2_-adrenergic action via allosteric sites in β_2_-adrenergic receptors (synergism). In conclusion, K_Ca_ channels and allosterism may be novel targets of bronchodilator therapy for diseases such as asthma and COPD.

## 1. Introduction

Since the functional antagonism between β_2_-adrenergic and muscarinic receptors regulates airway smooth muscle tone, investigation of this antagonism may play a key role for progressing bronchodilator therapy for asthma and chronic obstructive pulmonary disease (COPD) [1,2,3,4]. Airway smooth muscle generates tension via Ca^2+^ signaling, which consists of an increase in intracellular concentration of Ca^2+^ (Ca^2+^ dynamics) and an increase in sensitivity to intracellular Ca^2+^ (Ca^2+^ sensitization) [5,6,7,8,9,10,11]. Potassium ion (K^+^) channels also contribute to smooth muscle tone via Ca^2+^ signaling [12,13,14,15]. Ca^2+^ dynamics may be associated with GTP-binding (G) proteins (G_s_, G_i_)/large-conductance Ca^2+^-dependent K^+^ (K_Ca_) channels/L-type voltage-dependent Ca^2+^ (VDC) channel linkage, leading to Ca^2+^-dependent contraction [1,2,16,17]. Hence, K_Ca_ channels may be responsible for therapeutic strategies for various diseases, including asthma and COPD [4,18,19,20].

The combination of β_2_-adrenergic receptor agonists with muscarinic receptor antagonists markedly enhances relaxant effects on muscarinic contraction [4,11,21]. This effect is due to crosstalk between β_2_-adrenergic and muscarinic receptors (G protein-coupled receptors: GPCRs), and is also involved in Ca^2+^ signaling mediated by K_Ca_ channels [4,11] and protein kinase C (PKC) [22,23,24]. The recent COPD guidelines state that a combination of bronchodilators of different pharmacological classes may improve effectiveness and decrease the risk of adverse reactions, compared to increasing the dose of a single bronchodilator [25]. There is a pharmacological rationale for combining β_2_-adrenergic receptor agonists and muscarinic receptor antagonists as a bronchodilator therapy for asthma and COPD [4,11,21,24,26]. Clinical trials have indicated that this combination therapy is beneficial to these diseases [27,28,29].

Both orthosteric and allosteric sites exist on GPCRs, and the effects of an agent (an orthosteric ligand) are influenced (potentiated or inhibited) via allosteric effects. An agonist binds to GPCRs as an allosteric modulator at an allosteric site, which is topographically distinct from an orthosteric site, leading to changes in response to an agent via an alteration in receptor conformation. Hence, allosteric GPCR modulation provides pharmacological characteristics, such as affinity, efficacy, and agonism [30,31]. Since allosteric effects may occur in the interaction mediated by ligands for GPCRs [30,31,32], allosteric GPCR modulation may contribute to the synergistically relaxant effects of the combination of β_2_-adrenergic receptor agonists with muscarinic receptor antagonists against muscarinic contraction in airway smooth muscle. As shown in a recent report [4,15], allosteric GPCR modulation is involved in the alteration of responsiveness to β_2_-adrenergic action against muscarinic contraction. However, little is known about the particular role of allosterism in the functional antagonism between β_2_-adrenergic and muscarinic receptors.

This study was designed to the determine involvement of K_Ca_ channel/VDC channel linkages in the synergistic effects between β_2_-adrenergic receptor agonists and muscarinic receptor antagonists. Moreover, the role of allosteric GPCR modulation in this synergism was investigated using physiological techniques in airway smooth muscle.

## 2. Results

### 2.1. Synergism in the Combination of β_2_-Adrenergic Receptors with Muscarinic Receptor Antagonists

**Figure 1:**
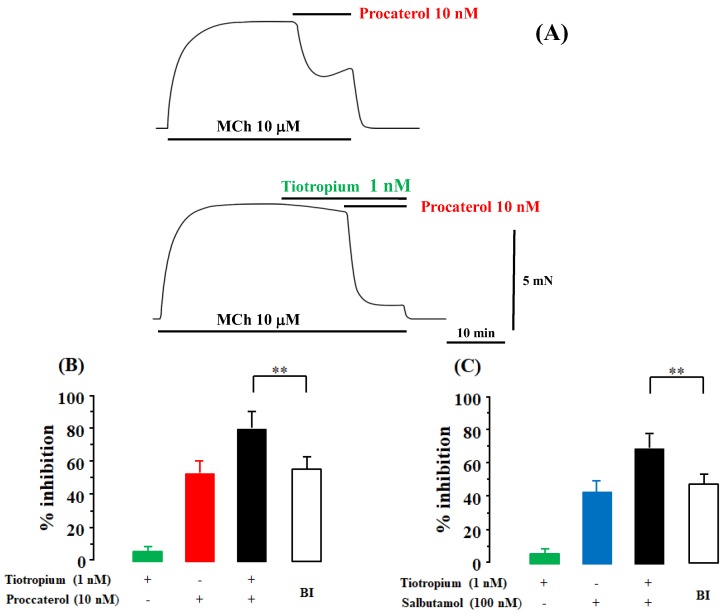
Synergistic effects of combination of β_2_-adrenergic receptor agonists with muscarinic receptor antagonists in airway smooth muscle. (**A**) Typical results of the inhibitory effect of procaterol (10 nM) in the absence (upper side) and presence (lower side) of tiotropium (1 nM) against methacholine (MCh, 10 μM)-induced contraction; (**B**) Values of percent inhibition of tiotropium (1 nM), procaterol (10 nM), and the combination of these two agents; (**C**) Values of percent inhibition of tiotropium (1 nM), salbutamol (100 nM), and the combination of these two agents. BI: the values of percent inhibition predicted by the Bliss independence theory, **: *p* < 0.01.

Tiotropium (1 nM) caused a modest inhibition (5.9 ± 1.3%, *n* = 8) [95% CI: 4.81–6.99] of methacholine (MCh, 10 μM)-induced contraction (Figure 1A,B). Procaterol (10 nM) caused a 52.2 ± 6.9 percent inhibition [95% CI: 46.43–57.97] of MCh (10 μM)-induced contraction (*n* = 8) (Figure 1A,B). When procaterol (10 nM) was applied to the tissues pre-contracted by MCh (10 μM) in the presence of tiotropium (1 nM), the inhibitory effects of the combination of procaterol and tiotropium were markedly enhanced (Figure 1A), and values of percent inhibition were increased to 80.8 ± 9.0% [95% CI: 73.27–88.33] (*n* = 8, Figure 1B). Under this experimental condition, the values of percent inhibition were considerably greater than the values of percent inhibition predicted by the Bliss independence (BI) theory (55.1 ± 5.9%, 95% CI: 50.17–60.03, *n* = 8, *p* < 0.01; Figure 1B). Similar results were observed for salbutamol and tiotropium. Salbutamol (100 nM) caused a 44.1 ± 6.2 percent inhibition [95% CI: 38.92–49.28] of MCh (10 μM)-induced contraction (*n* = 6, Figure 1C). When salbutamol (100 nM) was applied in the presence of tiotropium (1 nM), the inhibitory effects of the combination of salbutamol and tiotropium were markedly enhanced, and values of percent inhibition increased to 69.7 ± 6.6% [95% CI: 64.18–75.22] (*n* = 8, Figure 1C). Under these experimental conditions, the values of percent inhibition were considerably higher than the values predicted by the BI theory (48.1 ± 5.7%, 95% CI: 43.33–52.87, *n* = 8, *p* < 0.01; Figure 1C).

### 2.2. Role of G Protein/Ca^2+^-Activated K^+^ Channel Linkage in the Synergistic Effects

**Figure 2:**
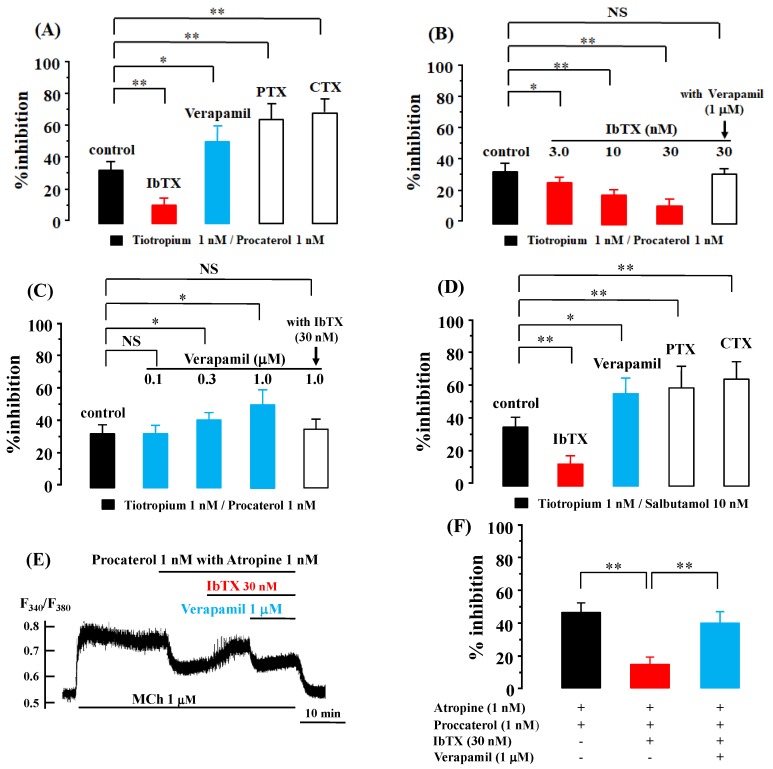
Mechanisms of synergistic effects of the combination of β_2_-adrenergic receptor agonists with muscarinic receptor antagonists. (**A**) Values of percent inhibition of procaterol (1 nM) with tiotropium (1 nM) against MCh (1 μM)-induced contraction (control) in the presence of IbTX (30 nM), or verapamil (1 μM), and after incubation with pertussis toxin (1 μg/mL, PTX) and cholera toxin (2 μg/mL, CTX) for six hours; (**B**) Values of percent inhibition of procaterol with tiotropium against MCh-induced contraction in the presence of IbTX (3.0–30 nM) without/with verapamil (1 μM); (**C**) Values of percent inhibition of procaterol with tiotropium against MCh-induced contraction in the presence of verapamil (0.1–1.0 μM) without/with IbTX (30 nM). (**D**) Values of salbutamol (10 nM) with tiotropium (1 nM) against MCh (1 μM)-induced contraction under the same experimental conditions as 2A; (**E**) A typical record of F_340_/F_380_ demonstrating the effect of IbTX (30 nM) and verapamil (1 μM) on a combination of procaterol (1 nM) with atropine (1 nM) in the presence of MCh (1 μM); (**F**) Values of percent inhibition of F_340_/F_380_ in MCh (1 μM)-induced contraction under the condition of procaterol with atropine in the absence and presence of IbTX and verapamil. MCh: methaxholine, IbTX: iberiotoxin, NS: not significant, *: *p* < 0.05, **: *p* < 0.01.

When procaterol (1 nM) was combined with tiotropium (1 nM), MCh (10 μM)-induced contraction was attenuated by 33.7 ± 5.3% [95% CI: 29.91–37.49] (*n* = 10, Figure 2A). In the presence of iberiotoxin (IbTX, 30 nM), the effects of this combination of procaterol (1 nM) with tiotropium (1 nM) were markedly attenuated to 13.2 ± 4.4% [95% CI: 9.52–16.88] (*n* = 8, *p* < 0.01, Figure 2A). This inhibitory effect of IbTX was concentration-dependent; in the presence of IbTX (3.0 and 10 nM) the effects of this combination of agents were attenuated to 26.7 ± 3.8% [95% CI: 23.52–29.88] (*p* < 0.05) and 19.0 ± 4.3% [95% CI: 15.40–22.60] (*p* < 0.01), respectively (each *n* = 8, Figure 2B). The inhibitory effect of IbTX (30 nM) was reversed to 32.8 ± 3.9% [95% CI: 29.54–36.06] (*n* = 8, not significant) in the presence of verapamil (1 μM) (Figure 2B). In contrast, the inhibitory effects of procaterol with tiotropium were markedly augmented to 52.9 ± 9.4% [95% CI: 45.04–60.76] in the presence of verapamil (1 μM) (*n* = 8, *p* < 0.05, Figure 2A). The stimulatory effect of verapamil was concentration-dependent; in the presence of verapamil (0.1 and 0.3 μM) the effects of this combination of these agents were augmented to 34.5 ± 5.3% [95% CI: 30.07–38.98] (not significant) and 42.8 ± 4.7% [95% CI: 38.87–46.73] (*p* < 0.05), respectively (each *n* = 8, Figure 2C). The effect of verapamil was decreased to 36.1 ± 6.0% [95% CI: 31.08–41.12] (*n* = 8, not significant) in the presence of IbTX (30 nM) (Figure 2C). Moreover, after the tissues were incubated with pertussis toxin (PTX, 1 μg/mg) to suppress G_i_ activity or with cholera toxin (CTX, 2 μg/mL) to increase G_s_ activity for six hours, the inhibitory effects of this combination of these two agents were markedly improved to 66.6 ± 9.7% [95% CI: 56.42–76.78] (*n* = 6, *p* < 0.01) and 70.8 ± 8.5% [95% CI: 61.88–79.72] (*n* = 6, *p* < 0.01), respectively (Figure 2A). Similar results were observed for salbutamol and tiotropium. The combination of salbutamol (10 nM) with tiotropium (1 nM) caused a 36.4 ± 6.1 percent inhibition of MCh (10 μM)-induced contraction [95% CI: 31.30–41.51] (*n* = 8, Figure 2D). This effect of a combination of these two agents was attenuated to a 14.8 ± 4.8 percent inhibition [95% CI: 9.76–19.84] (*n* = 6, *p* < 0.01) in the presence of IbTX (30 nM); in contrast, this was increased to a 56.4 ± 7.3 percent inhibition (*n* = 6, *p* < 0.05) in the presence of verapamil (1 μM) (Figure 2D). Pre-exposure to PTX and CTX for six hours also enhanced the inhibitory effects of salbutamol with tiotropium. Values of percent inhibition were increased to 60.8 ± 8.1% [95% CI: 52.30–69.30] (*n* = 6, *p* < 0.01) and 65.6 ± 6.6% [95% CI: 58.67–72.53] (*n* = 6, *p* < 0.01), respectively (Figure 2D). When procaterol (1 nM) with atropine (1 nM) was applied to the Fura-2 loaded tissues pre-contracted by MCh (1 μM), F_340_/F_380_ (an indicator of concentration of intracellular Ca^2+^) was decreased by 46.5 ± 5.2% [95% CI: 38.23–54.77] (*n* = 8, *p* < 0.01, Figure 2C,D). IbTX reduced the percent inhibition of F_340_/F_380_ induced by procaterol with atropine to 14.8 ± 4.4% [95% CI: 6.44–23.16] (*n* = 4, *p* < 0.01), on the other hand, IbTX with verapamil augmented the values of F_340_/F_380_ to 38.5 ± 5.9% [95% CI: 27.26–49.74] (*n* = 4, *p* < 0.01, Figure 2E,F).

### 2.3. Role of Orthosteric Sites in the Effects of Muscarinic Receptor Antagonists

**Figure 3:**
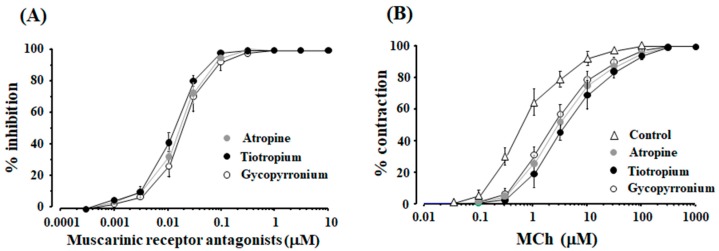
Effects of a muscarinic receptor antagonist on muscarinic contraction via orthosteric sites. (**A**) Concentration-inhibition curves for atropine, tiotropium and glycopyrronium against MCh (10 μM); (**B**) Concentration-response curves for MCh (control) in the absence and presence of atropine, tiotropium and glycopyrronium (each 10 nM). MCh: methaxholine.

When atropine, tiotropium and glycopyrronium (0.0003–10 μM) were cumulatively applied to the tissues pre-contracted with MCh (1 and 10 μM), these agents inhibited MCh-induced contraction in a concentration-dependent manner, and complete inhibition was observed under these experimental conditions (Table 1). When the concentration of MCh was increased to 10 μM, these agents also caused a concentration-dependent inhibition, and complete relaxation was also observed at 0.3 μM of each agent (Figure 3A). Moreover, the complete relaxation was not attenuated even when the concentration of these agents were increased to more than 0.3 μM (Figure 3A). The concentration-inhibition curves for these agents against MCh (1 and 10 μM), the values of EC_50_ and the maximal effect of these curves for these agents under each experimental condition are summarized in Table 1. On the other hand, when MCh was cumulatively applied to the tissues, the value of EC_50_ was 0.6 ± 0.3 μM [95% CI: 0.26–0.68] (*n* = 6), and maximal response was observed at 100 μM (Figure 3B). In the concentration-response curves for MCh in the presence of atropine, tiotropium and glycopyrronium at each 10 nM, values of EC_50_ increased to 2.8 ± 0.9 [95% CI: 1.86–3.75] (*n* = 6), 3.9 ± 1.2 [95% CI: 2.64–5.16] (*n* = 6), 2.3 ± 0.7 μM [95% CI: 1.57–3.04] (*n* = 6), respectively (not significant), and the maximal contraction was not diminished under this experimental condition (Figure 3B).

### 2.4. Role of Allosteric Sites in the Effects of β_2_-Adrenergic Receptor Agonists

**Figure 4:**
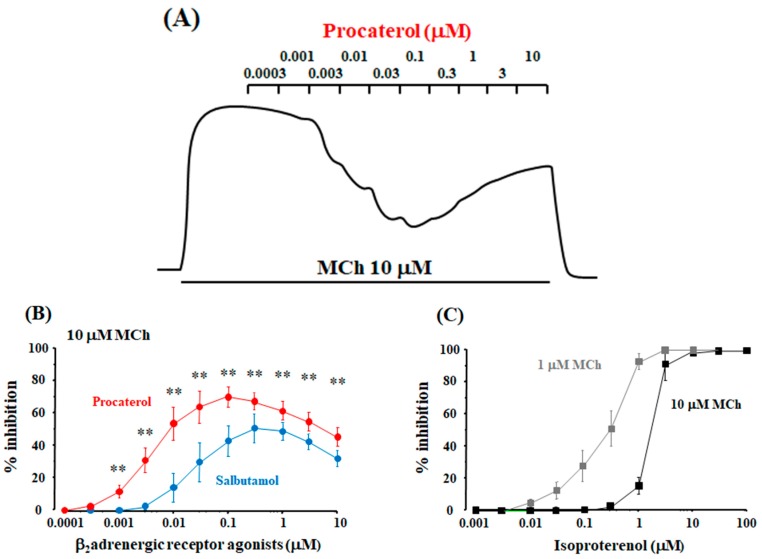
Effects of β_2_-adrenergic receptor agonists on muscarinic contraction via allosteric effects (efficacy modulation) in β_2_-adrenergic receptors. (**A**) A typical example of continuous recording of cumulative applications of procaterol to the tissues pre-contracted by 10 μM MCh; (**B**) Concentration-inhibition curves for procaterol and salbutamol against contraction induced by MCh (10 μM); (**C**) Concentration-inhibition curves for isoproterenol against contraction induced by MCh (1 and 10 μM). MCh: methacholine. **: *p* < 0.01.

**Figure 5:**
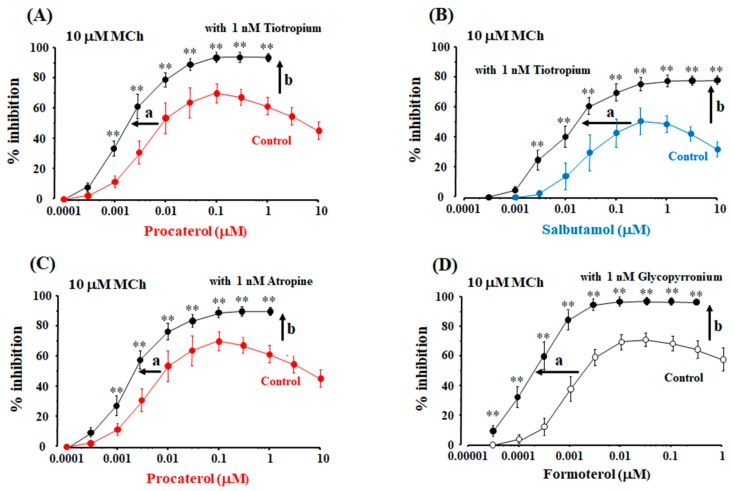
Synergistic effects of a combination of β_2_-adrenergic receptor agonists with muscarinic receptor antagonists against muscarinic contraction via allosteric effects (affinity and efficacy modulation). Concentration-inhibition curves for procaterol (**A**) and salbutamol (**B**) against MCh (10 μM)-induced contraction in the absence and presence of tiotropium (1 nM); (**C**) Concentration-inhibition curves for procaterol against MCh (10 μM)-induced contraction in the absence and presence of atropine (1 nM); (**D**) Concentration-inhibition curves for formoterol against MCh (10 μM)-induced contraction in the absence and presence of glycopyrronium (1 nM). MCh: methacholine, (**a**): affinity, (**b**): efficacy, **: *p* < 0.01.

When procaterol and salbutamol were cumulatively applied to the tissues pre-contracted with MCh (1 and 10 μM), the maximal inhibition was observed for each experimental condition; the inhibitory effects of these agents were attenuated in a concentration-dependent manner at higher concentrations than the concentrations that produce the maximal effects (Figure 4A,B). Similar results were observed in the cumulative application of formoterol to MCh (10 μM)-induced contraction (Figure 5D). Procaterol caused almost complete relaxation (100% inhibition) of MCh (1 μM)-induced contraction, whereas, complete inhibition did not occur in other experimental conditions (Figure 4A,B and Figure 5D). On the other hand, when isoproterenol was cumulatively applied to the tissues pre-contracted with MCh (1 and 10 μM), it caused complete inhibition against MCh-induced conditions; moreover, the inhibitory effects of isoproterenol were not attenuated at the higher concentrations that produce the maximal effects, which is different from procaterol, salbutamol and formoterol (Figure 4C and Figure 5D). The concentration-inhibition curves for these agents against MCh (1 and 10 μM)-induced contraction, the values of EC_50_ and the maximal inhibition are summarized in Table 2.

### 2.5. Role of Allosteric Effects in the Synergistic Effects of β_2_-Adrenergic Receptor Agonists with Muscarinic Receptor Antagonists

When tiotropium, atropine, and gltopyrronium (each 1 nM) were exposed to the tissues pre-contracted with MCh (10 μM) for 120 min, the maximal inhibition of tiotropium was 8.2 ± 2.1% (*n* = 10) [95% CI: 2.22–14.18], 6.8 ± 1.9% (*n* = 10) [95% CI: 1.39–12.20] and 6.2 ± 1.4% [95% CI: 2.22–10.18] (*n* = 10), respectively, for this extended period. Although these agents were slow to act, the time-dependent effects were modest under this experimental condition. Procaterol and salbutamol were cumulatively applied to the tissues pre-contracted with MCh (10 μM) with tiotropium (1 nM). In the presence of tiotropium (1 nM), concentration-inhibition curve for procaterol against MCh (10 μM)-induced contraction were significantly dissociated from the curves for procaterol in the absence of tiotropium. In the curves for procaterol with tiotropium, the values of EC_50_ were significantly decreased to 1.6 ± 0.4 nM [95% CI: 1.27–1.91] (*n* = 8, *p* < 0.01, Figure 5A). Moreover, in the curves for procaterol with tiotropium, maximal inhibition of the curves for procaterol was markedly increased to 94.3 ± 4.6% [95% CI: 90.45–98.15] (*n* = 8, *p* < 0.01; Figure 5A). The maximal effects of procaterol were not attenuated at higher concentrations than those concentrations than produce the maximal effects, unlike the effects of procaterol without tiotropium. Similar results were observed for salbutamol with tiotropium. Concentration-inhibition curves for salbutamol with tiotropium (1 nM) against MCh (10 μM) were also significantly dissociated from the curves for salbutamol without tiotropium. In the curves for salbutamol with tiotropium, the values of EC_50_ were significantly decreased to 12.8 ± 6.6 nM [95% CI: 7.22–18.12] (*n* = 8, *p* < 0.01; Figure 5B). Maximal inhibition of the curves for salbutamol with tiotropium increased to 78.3 ± 8.6% [95% CI: 71.11–85.49]; the maximal effects of salbutamol were also not attenuated at higher concentrations than those concentrations that produce the maximal effects (*n* = 8, *p* < 0.01; Figure 5B). In the concentration-inhibition curves for procaterol with atropine (1 nM) against MCh (10 μM), the values of EC_50_ were significantly decreased to 2.0 ± 0.8 nM [95% CI: 1.16–2.84] (*n* = 6, *p* < 0.01; Figure 5C), and the maximal inhibition was increased to 91.3 ± 6.4% [95% CI: 84.58–98.02] (*n* = 6, *p* < 0.01; Figure 5C). In the concentration-inhibition curves for formoterol with glycopyrronium (1 nM) against MCh (10 μM), the values of EC_50_ were significantly decreased to 0.20 ± 0.04 nM [95% CI: 0.16–0.24] (*n* = 6, *p* < 0.01; Figure 5D), and the maximal inhibition was increased to 96.4 ± 3.4% [95% CI: 92.81–99.97] (*n* = 6, *p* < 0.01; Figure 5D). The maximal effect was not attenuated at higher concentrations than those concentrations that produced the maximal effect (Figure 5D), similar to the combination of procaterol and salbutamol with tiotropium.

### 2.6. Possible Mechanisms of cAMP-Independent Processes in the Synergistic Effect

**Figure 6:**
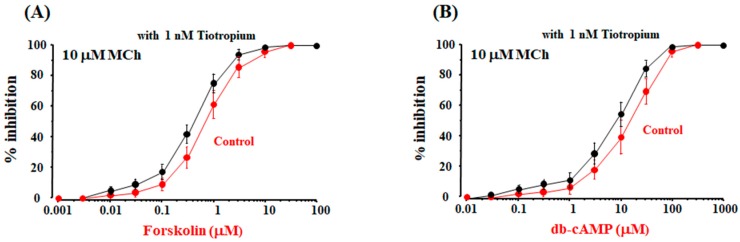

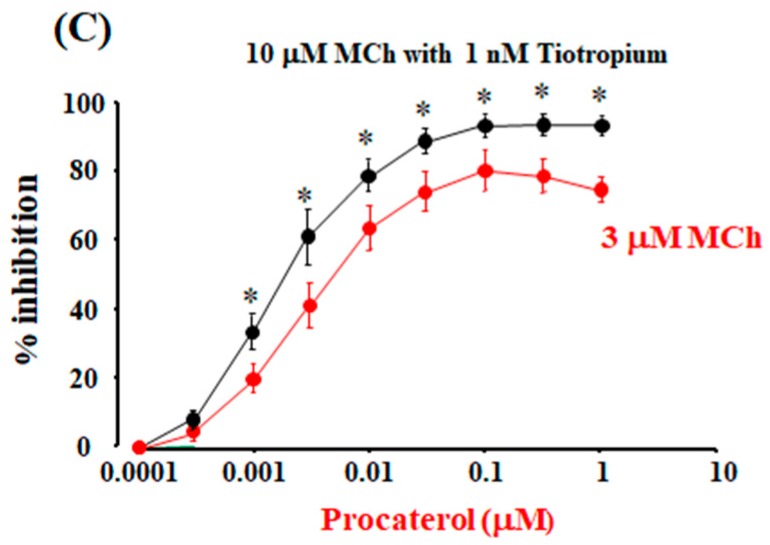
The synergistic effect is not due to bypassing β_2_-adrenergic receptors and blocking muscarinic receptors. Concentration-inhibition curves for forskolin (**A**) and db-cAMP (**B**) in the absence and presence of tiotropinm (1 nM). (**C**) Concentration-inhibition curves for procaterol against MCh (3 μM)-induced contraction. The curve for procaterol against MCh (10 μM) with tiotropium (1 nM) is superimposed (ref. Figure 5A). *: *p* < 0.05.

Forskolin and db-cAMP, which are independent of β_2_-adrenergic receptors, caused a concentration-dependent suppression of MCh (10 μM)-induced contraction, and complete relaxation (100% inhibition) was observed for each experimental condition. The maximal effects of these agents were not attenuated at higher concentrations than those concentrations than produce the maximal effects in the absence and presence of tiotropium, unlike β_2_-adrenerigic receptor agonists. In the concentration-inhibition curves for forskolin and db-cAMP, EC_50_ was not significantly reduced in the present of tiotropium (1 nM) (Figure 6A,B), which is different to β_2_-adrenerigic receptor agonists. Under these experimental conditions, the values of EC_50_ were 0.69 ± 0.28 [95% CI: 0.40–0.98] and 0.41 ± 0.18 μM [95% CI: 0.22–0.60], respectively (*n* = 6, not significant, Figure 6A), and 10.4 ± 4.6 [95% CI: 5.57–15.23] and 8.1 ± 3.3 μM [95% CI: 4.64–11.57] (*n* = 6, not significant, Figure 6B), respectively.

### 2.7. Possible Involvement of Interaction between Muscarinic Receptor Antagonists and Allosteric Sites on β_2_-Adrenergic Receptors

When the concentration of MCh was decreased from 10 μM to 3 μM, MCh –induced contraction was attenuated by approximate 10% (Figure 3B). The effect of MCh (3 μM) was roughly equivalent to that of MCh (10 μM) with tiotropium (1 nM). In concentration-inhibition curves for procaterol against MCh (3 μM), the value of EC_50_ were 50.8 ± 15.4 nM [95% CI: 34.64–66.97] (*n* = 6, *p* < 0.05), and the value of the maximal inhibition were 80.3 ± 7.8% [95% CI: 72.11–88.49] (*n* = 6, *p* < 0.05, Figure 6C). The maximal effects of procaterol against MCh (3 μM) without muscarinic receptor antagonists were attenuated at higher concentrations in a concentration-dependent manner. The curves for procaterol against MCh (3 μM) were significantly different from those curves for procaterol with tiotropium (1 nM) against MCh (10 μM) (Figure 6C).

## 3. Discussion

This study used physiological methods to demonstrate that G proteins/K_Ca_ channel/VDC channel linkages are an essential process in the synergistic effects induced by crosstalk between β_2_-adrenergic and muscarinic M_2_ receptors. Furthermore, allosteric effects play a functionally important role in the alteration of the response to β_2_-adrenergic receptor agonists and muscarinic receptor antagonists via crosstalk between these two GPCRs, i.e., (1) reduced responsiveness to β_2_-adrenergic receptor agonists (partial agonism); and (2) enhanced responsiveness to the combination of β_2_-adrenergic receptor agonists with muscarinic receptor antagonists (synergism).

The combination of β_2_-adrenergic receptor agonists (procaterol, salbutamol) with a muscarinic receptor antagonist (tiotropium) synergistically enhanced the relaxant effects against muscarinic contraction (Figure 1), consistent with previous reports using other β_2_-adrenergic receptor agonists (indacaterol, formoterol) and muscarinic receptor antagonists (glycopyrronium, aclidinium) [4,11,24,33]. Moreover, the combination of atropine with procaterol and the combination of glycopyrronium with formoterol produced synergistic effects (Figure 5C,D). Therefore, this phenomenon is probably universal, not specific to an agent related to these two receptors.

In airway smooth muscle tone, Ca^2+^ signaling contributes to the intracellular processes in the downstream of these two GPCRs [6,8,34,35]. Suppression of K_Ca_ channel activity enhances muscarinic contraction with an increase in Ca^2+^ influx pass through VDC channels [17]. Ca^2+^ dynamics due to K_Ca_ channels [4,11] and Ca^2+^ sensitization due to PKC contributes to the synergistic effects between β_2_-adreneric receptor agonists and muscarinic receptor antagonists [24]. In this study, the effect of VDC channels was analyzed and whether the Ca^2+^ dynamics due to K_Ca_ channels is involved in this synergism was investigated. K_Ca_ channel activity is associated with the functional antagonism between β_2_-adrenergic and muscarinic receptors [1,2,4]. K_Ca_ channels are activated not only by protein kinase A (PKA) but also by G_s_, which is coupled to β_2_-adrenergic receptors [36,37]. In contrast, these channels are inhibited by G_i_, which is coupled to muscarinic M_2_ receptors [1,4,38]. The synergistic effects of β_2_-adrenergic receptor agonists with muscarinic receptor antagonists were markedly augmented when G_s_ was activated by cholera toxin and G_i_ is inhibited by pertussis toxin (Figure 2A,D). These synergistic effects may be caused by β_2_-adreneric receptor agonists/G_s_ protein/K_Ca_ channels’ stimulatory coupling and muscarinic M_2_ receptors/G_i_ protein/K_Ca_ channels’ inhibitory coupling. Moreover, the synergistic effects were inhibited by IbTX, an inhibitor of K_Ca_ channels, in a concentration-dependent manner (Figure 2A,B), indicating that activation of K_Ca_ channels is involved in this phenomenon. In contrast, the synergistic effects were augmented by verapamil, an inhibitor of VDC channels, in a concentration-dependent manner, indicating that inactivation of VDC channels is involved in this phenomenon (Figure 2A,C). IbTX and verapamil were antagonistic to each other in the regulation of synergistic effects (Figure 2B,C), indicating that coupling between K_Ca_ and VDC channels is involved in this phenomenon. IbTX inhibited a reduction in intracellular Ca^2+^ concentration induced by procaterol with atropine, in contrast, verapamil reversed this effect of IbTX (Figure 2E,F), indicating that suppression of K_Ca_ channels increases the concentration of intracellular Ca^2+^ via activation of VDC channels (Ca^2+^ dynamics due to K_Ca_ channels/VDC channels coupling). Activation of K_Ca_ channels causes membrane hyperpolarization, which deactivates VDC channels. This channel coupling enhances relaxation of airway smooth muscle [2,17]. Therefore, Ca^2+^ dynamics via these G proteins/K_Ca_ channel/VDC channel processes may contribute to the synergistic action (Figure 2). The functional antagonism to β_2_-aderenergic receptors also may be mediated by muscarinic M_2_ receptors [39,40,41].

**Figure 7:**
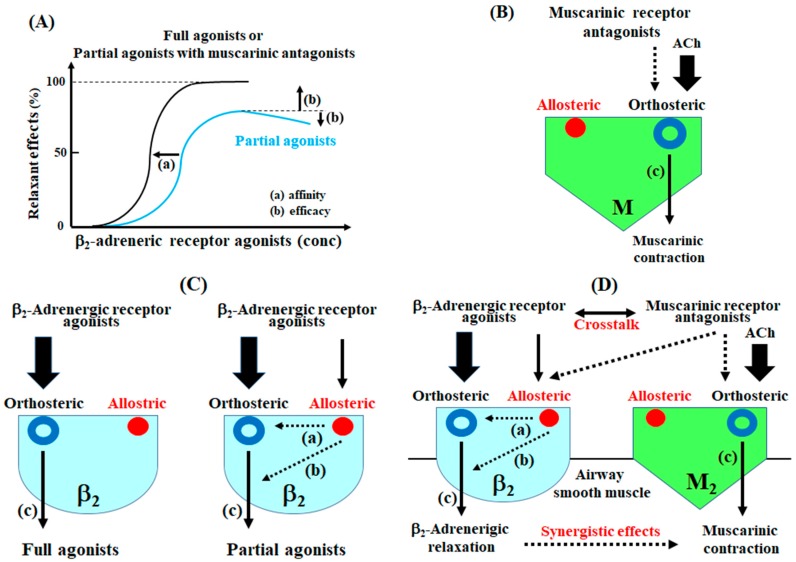
Involvement of allosteric effects (affinity and efficacy modulation) in response to β_2_-adrenergic receptor agonists and muscarinic receptor antagonists against muscarinic contraction. (**A**) A schema of affinity and efficacy modulation via allosteric sites shown in concentration-response curves for β_2_-adrenergic receptor agonists against muscarinic contraction; (**B**) Muscarinic receptor antagonists inhibit muscarinic action via acting on orthosteric sites on muscarinic receptors, independent of allosteric sites; (**C**) Full β_2_-adrenergic receptor agonists do not act on allosteric sites. In contrast, partial β_2_-adrenergic receptor agonists act not only on orthosteric sites, but also on allosteric sites on these receptors, and these agonists reduce the signal capacity (intrinsic efficacy) of an orthosteric ligand via efficacy modulation induced by operating upon allosteric sites; (**D**) Muscarinic receptor antagonists act on allosteric sites of β_2_-adrenergic receptors, and as a result affinity and efficacy of β_2_-adrenergic receptor agonists are enhanced, leading to the synergistically relaxant action of the combination of β_2_-adrenergic receptor agonists with muscarinic receptor antagonists via crosstalk of these two receptors. M: muscarinic receptors, β_2_: β_2_-adrenergic receptors, M_2_: muscarinic M_2_ receptors. (**a**): affinity, (**b**): efficacy, (**c**): signal capacity (intrinsic efficacy). Arrows: activation, dotted arrows: inhibition.

Allosteric affinity and efficacy modulation to muscarinic receptors and β_2_-adenergic receptors was examined using the concentration-inhibition curves for muscarinic receptor antagonists and β_2_-adrenergic receptor agonists against muscarinic contraction (Figure 3 and Figure 4) [4,42,43], and the intrinsic efficacy of muscarinic receptor antagonists and β_2_-adrenergic receptor agonists was determined under these experimental conditions (Table 1 and Table 2). Allosteric modulators alter association or dissociation rates of orthosteric ligands (affinity modulation) via acting allosteric sites. Allosteric effects operate intracellular responses and alter the signaling capacity (intrinsic efficacy) of orthosteric ligands (efficacy modulation) [30,31,32]. When agents act on their specific GPCRs at orthosteric sites without allosteric sites, the maximal response to agents as orthosteric ligands is not attenuated (Figure 7A,B) because an allosteric modulation does not occur (full agonism). In the functional antagonism (crosstalk) between β_2_-adrenergic and muscarinic receptors, when agents act upon their specific GPCRs at both orthosteric and allosteric sites, partial agonism of β_2_-adrenergic receptors is generated because response to agents as orthosteric ligands is altered by allosteric efficacy modulation (Figure 7A,C) [4,43].

To determine the involvement of orthosteric and allosteric sites in the relaxant action of muscarinic receptor antagonists against muscarinic contraction, affinity and efficacy modulation of these antagonists were examined using concentration-inhibition curves for these antagonists on a muscarinic receptor agonist. Muscarinic receptor antagonists, including atropine, tiotropium, and glycopyrroium, caused the maximal effect (complete inhibition) of muscarinic contraction, and the maximal effect was not attenuated even when higher concentrations of these antagonists were applied (Figure 3A, Table 1). Moreover, in the concentration-response curves for MCh with these antagonists, the values of EC_50_ were markedly increased without reducing the maximal response (Figure 3B). Since muscarinic receptor antagonists competitively inhibit the effects of a ligand on the receptor, these agents do not alter the signal capacity of orthosteric ligands via binding to allosteric sites [42,43]. Hence, muscarinic receptor antagonists do not cause efficacy modulation against orthosteric effects in muscarinic action, and allosteric sites on muscarinic receptors are not associated with muscarinic antagonism induced by these agents (Figure 7B) [42,43].

To determine the involvement of orthosteric and allosteric sites in the relaxant action of β_2_-adreneric receptor agonists against muscarinic contraction, affinity and efficacy modulation of these agonists were examined using concentration-inhibition curves for these agonists on a muscarinic receptor agonist. Isoproterenol completely antagonized muscarinic contraction, and the complete inhibition induced by isoproterenol was not attenuated (Figure 4C) at higher concentrations than those that produce maximal relaxation. Isoproterenol acts on orthosteric sites in β_2_-adrenergic receptors and does not act on allosteric sites in these receptors (Figure 7A,C). Hence, isoproterenol behaves as a full agonist. In contrast, procaterol, salbutamol and formoterol incompletely antagonize muscarinic contraction (Figure 4A,B and Figure 5D), similar to indacaterol and salmeterol [4,11,44,45]. A lack of complete inhibition by β_2_-adrenergic receptor agonists indicates a decrease in the signal capacity induced by efficacy modulation (an inhibition in response to orthosteric site via allosterism). At higher concentrations than those that produce the maximal relaxation, procaterol, salbutamol and formoterol caused a concentration-dependent contraction, unlike isoproterenol, suggesting that the primary effects (signal capacity) of these agents via orthosteric sites are reduced by efficacy modulation via binding to allosteric sites (Figure 7A,C). Therefore, in the β_2_-adrenergic receptor agonists, except isoproterenol, intrinsic efficacy is attenuated by stimulating allosteric sites, indicating that these agonists behave as allosteric modulators against β_2_-adrenergic receptors (partial agonists) (Figure 7A,B, Table 2) [4,11,44,45].

To determine the involvement of orthosteric and allosteric sites in this synergistic effect between β_2_-adreneric receptor agonists and muscarinic receptor antagonists, affinity and efficacy modulation of β_2_-adreneric receptor agonists with muscarinic receptor antagonists were examined using concentration-inhibition curves for these agonists on a muscarinic receptor agonist. Allosteric modulation occurs in the interaction mediated by ligands related to GPCRs [30,31,32]. When agents act not only on their specific GPCGs at orthosteric sites as othosteric ligands, but also on other GPCRs at allosteric sites, synergism may be generated via affinity and efficacy modulation due to allosterism [4,15]. The combination of β_2_-adrenergic receptor agonists with muscarinic receptor antagonists may synergistically inhibit muscarinic contraction via allosterism (affinity and efficacy modulation). In the concentration-inhibition curves for procaterol, salbutamol and formoterol with muscarinic receptor antagonists (tiotropium, atropine, glycopyrronuium), the values of EC_50_ were markedly decreased; the maximal effects of these β_2_-adrenergic receptor agonists were markedly augmented in each experimental condition (Figure 5). Muscarinic receptor antagonists may enhance affinity and efficacy of β_2_-adrenergic receptor agonists, leading to synergistic effects on muscarinic contraction (Figure 7A,D). However, this synergistic effect may be due to inhibition of muscarinic contraction induced by muscarinic receptor antagonists. To determine whether this phenomenon is involved in the synergistic effect, affinity and efficacy modulation of β_2_-adrenergic receptor agonists were examined using concentration-inhibition curves for these agents without muscarinic receptor antagonists under the condition of a lower concentration of MCh. MCh (10 μM)-induced contraction with 1 nM of atropine, tiotropium and glycopyrronium was roughly equivalent to MCh (3 μM)-induced contraction (Figure 3A,B). The concentration-inhibition curve for procaterol against MCh (3 μM)-induced contraction was no different from the curve for procaterol with tiotropium (1 nM) against MCh (10 μM)-induced contraction (Figure 6C). Therefore, this synergistic effect is created independently of the effects of muscarinic receptor antagonists on their receptors. Muscarinic receptor antagonists may operate not only upon orthosteric sites on muscarinic receptors, but also upon allosteric sites on β_2_-adrenergic receptors, and these antagonists enhance both affinity and efficacy to β_2_-adrenergic receptors; as a result, this synergism may be generated via crosstalk between the two GPCRs (Figure 7D).

To determine whether the downstream of β_2_-adrenergic receptor is involved in the synergistic effect, similar experiments were carried out using cAMP-related agents independent β_2_-adrenergic receptors. Forskolin and N6-dibutylyl cyclic AMP (db-cAMP), which are unrelated to β_2_-adrenergic receptors, did not cause synergistic effects in the presence of tiotropium (Figure 6A,B), which is different toβ_2_-adrenergic receptor agonists with muscarinic receptor antagonists (Figure 5). In human bronchial smooth muscle, intracellular concentration of cAMP is associated with the synergism between β_2_-adrenergic receptor agonists and muscarinic receptor antagonists [46]. However, adenylyl cyclase/cAMP/PKA processes may not directly contribute to the synergistic effects between these two agents, because forskolin and db-cAMP caused a modest effect on this synergism (Figure 6A,B). This result is similar to the phenomenon that G_s_ is more potent in K_Ca_ channel activation than PKA; K_Ca_ channels are markedly activated by G_s_ independent of PKA [37]. The β_2_-adrenergic receptor/G_s_ pathway, and muscarinic M_2_ receptor/G_i_ pathway may be essential to the synergism between these two agents.

## 4. Materials and Methods

### 4.1. Tissue Preparation and Tension Records

The methods were similar to those described previously [2,47,48]. Male Hartley guinea pigs (250–350 g) were killed by injection of an overdose of anesthetics (150 mg/kg pentobarbital i.p.), and tracheas were excised from the animals. The tracheal ring with three segments of cartilage was removed and placed horizontally in a 2-mL organ bath for isometric tension recording. Tracheal smooth muscle strips were incubated with 15 μM Fura-2/AM for about 2 h at room temperature (22–24 °C). The non-cytotoxic detergent Pluronic F-127 (0.01% *w*/*v*) was added to increase the solubility of Fura-2/AM. The intensities of excitation fluorescence at 340 nm and 380 nm were measured after background subtraction by exposure of the mucosal side to excitation light. The ratio of F_340_ to F_380_ (F_340_/F_380_) was used as a relative indicator of intracellular Ca^2+^ concentration [6,7,8]. Strips were perfused with the normal bathing solution at a constant flow rate of 3.0 mL/min throughout the experiments. The normal bathing solution was composed of NaCl (137 mM), KHCO_3_ (5.9 mM), CaCl_2_ (2.4 mM), MgCl_2_ (1.2 mM), and glucose (11.8 mM), bubbled with a mixture of 99% O_2_ and 1% CO_2_ (pH 7.4). After equilibrating the preparation in normal bathing solution, the experiments were started, and the temperature of the organ bath was maintained at 37 °C. The resting tone was abolished by the addition to indomethacin (1 μM) throughout the experiments. All animal procedures in this study were approved by the Animal Care and Use Committee, Kindai University Faculty of Medicine (identification code: KAME-22-007, 24 February 2011).

### 4.2. Analysis of Synergistic Effect

The synergistic effects of the combination of procaterol or salbutamol with tiotropium were evaluated using the Bliss independence (BI) theory. This model assumes that two or more agents act independently, with different modes and sites of action (Greco et al., 1995; Goldoni and Johansson, 2007), and is expressed by the following equation: E(x, y) = E(x) + E(y) − E(x) × E(y) where E is the fractional effect, and x and y are the doses of two compounds in a combination experiment. If the combined experimental value is higher than the expected value, the interaction is synergistic. If it is lower, the interaction is antagonistic [24,49,50].

### 4.3. Experimental Protocols

Concentration-response curves were plotted by the cumulative addition of agents at 10 min for each concentration. To examine the effect of inhibited (uncoupled to the muscarinic M_2_ receptor) G_i_, the inhibitory G protein of adenylyl cyclase, via adenosine diphosphate (ADP) ribosylation of the α-subunit of G_i_ protein, the strips of tracheal smooth muscle were incubated with 1 μg/mL petussis toxin (PTX) for 6 h and then the PTX was washed out. To examine the effects of activated G_s_, the stimulatory G protein of adenylyl cyclase coupled to β_2_-adrenergic receptors, via ADP ribosylation of the α-subunit of G_s_ protein, the tissues were incubated with 2 μg/mL cholera toxin (CTX) for 6 h and then the CTX was washed out. The involvement of K_Ca_ channels and VDC channels was examined by application of iberiotoxin (IbTX), a potent selective inhibitor of K_Ca_ channels, and verapamil, a selective inhibitor of VDC channels. To examine allosteric effects (alteration of intrinsic efficacy of an orthosteric ligand: efficacy modulation) in the relaxant action of β_2_-adrenergic receptor agonists on muscarinic contraction were cumulatively applied to the tissues pre-contracted by methacholine (MCh, 1 and 10 μM). Moreover, to examine allosteric effects (affinity and efficacy modulation) in the relaxant action of the combination of β_2_-adrenergic receptor agonists with muscarinic receptors antagonists against muscarinic contraction, procaterol and salbutamol were cumulatively applied to the tissues pre-contracted by MCh (10 μM) in the absence and presence of muscarinic receptor antagonists (1 nM). The concentration-inhibition curves for β_2_-adrenergic receptor agonists were analyzed for each experimental condition. Time-matched control tissues were treated similarly to the test tissues, but exposed continuously to the normal bathing solution (sham incubation) instead of agents. Procaterol, salbutamol, and formoterol were used as β_2_-adrenergic receptor agonists. Atropine, tiotropium, and glycopyrronium were used as muscarinic receptor antagonists. Forskolin (a direct activator of adenylyl cyclase) and N6-dibutylyl cyclic AMP (db-cAMP, an analog of cAMP) were used as cAMP-related agents independent of β_2_-adrenergic receptors.

### 4.4. Materials

MCh, indomethacin, procaterol, salbutamol, isoproterenol, formoterol, atropine, and glycopyrronium were obtained from Wako pure Chemical Industries (Osaka, Japan). IbTX was obtained from Peptide Institute (Osaka, Japan). Tiotropium, verapamil, PTX, CTX, forskolin, db-cAMP and indomethacin were obtained from Sigma (St. Louis, MO, USA).

### 4.5. Statistical Analysis

All data are expressed as the mean ± standard deviation (SD) with 95% confidence interval (CI). The response to an agent is described as a percentage of the maximal response. The effect of relaxant agents on MCh-induced contraction is expressed as percent inhibition by taking the control response to MCh (1 or 10 μM) for each experimental condition as 100%. The response to MCh was described as the percent contraction by taking the maximal contraction under the control condition as 100%. Values of the concentration of agents that produced a 50% response (EC_50_) of contraction were determined from the linear portion of each concentration-response curve. Statistical significance was assessed by unpaired Student’s *t*-test, one-way analysis of variance and the Dunnett test. A probability below 0.05 (*p* < 0.05) was considered to be significant.

## 5. Conclusions

**Figure 8:**
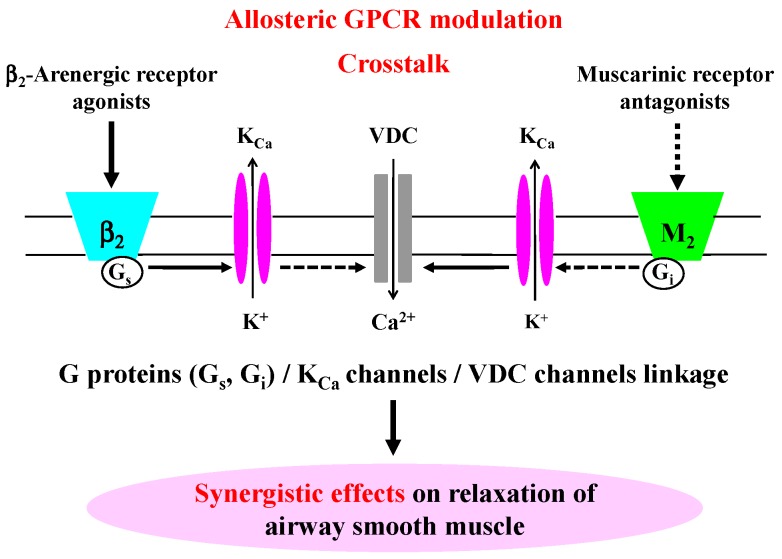
Possible clinical relevance of the synergistically relaxant effects between β_2_-adrenergic receptor agonists and muscarinic receptor antagonists. This synergism is caused by crosstalk based on allosteric GPCR modulation via G proteins (G_i_, G_s_)/K_Ca_ channel/VDC channel processes. Since this synergism causes greater bronchodilation, combining two agents may be beneficial to therapy for asthma and COPD. GPCR: G protein-coupled receptor, G_i_: the inhibitory G protein of adenylyl cyclase coupled to M_2_ receptors, G_s_: the stimulatory G protein of adenylyl cyclase coupled to β_2_-adrenergic receptors, K_Ca_: large-conductance Ca^2+^-activated K^+^ channels, VDC: L-type voltage-dependent Ca^2+^ channels. Arrows: activation, dotted arrows: inhibition.

Combining of β_2_-adrenergic receptor agonists with muscarinic receptor antagonists causes a synergistic inhibition against muscarinic contraction of airway smooth muscle. The G proteins/K_Ca_ channel/VDC channel linkage is a key molecule for this synergism, and allosteric GPCRs modulation induced by crosstalk between these two receptors plays a fundamental role in this phenomenon (Figure 8). Observations obtained from guinea pig trachea may have limited clinical relevance. However, muscarinic and β_2_-adrenergic action is not so different, not only between guinea pig trachea [4,11,24] and human bronchus [46], but also between guinea pig [47] and human trachea [48]. K_Ca_ channels are densely distributed on the airway smooth muscle in various mammals including humans; the electrical characteristics of these channels are no different between these species [51,52]. These results provide evidence that inhalation of a combined β_2_-adrenergic receptor agonist and a muscarinic antagonist causes greater bronchodilation than monotherapy in asthma and COPD [25,27,28,29]. The K_Ca_ channels/VDC channels coupling may also be responsible for β_2_-adrenergic desensitization [53] and airway remodeling [54]. Hence, elucidation of the mechanisms of this synergism (crosstalk) may contribute to the development of novel bronchodilators for asthma and COPD [4,11,55].

## Figures and Tables

**Table 1:** Pharmacological characteristics of muscarinic receptor antagonists.

Muscarinic Receptor Antagonists	EC_50_ (nM)				Emax (%)	
	MCh				MCh	
	1 μM (*n* = 6)		10 μM (*n* = 8)		1 μM (*n* = 6)	10 μM (*n* = 8)
	Mean ± S.D.	95% CI	Mean ± S.D.	95% CI	Mean ± S.D.	Mean ± S.D.
Tiotropium	3.3 ± 0.5	2.78–3.82	12.5 ± 3.2 *	9.82–15.18	100	100
Atropine	5.7 ± 1.1	4.55–6.86	17.5 ± 5.9 *	12.57–22.43	100	100
Glycopyrronium	8.2 ± 1.1	7.05–9.36	19.2 ± 7.1 *	13.26–25.14	100	100

* *p* < 0.05 vs. response to 1 μM MCh; Emax: maximal percent inhibition; 95% CI: 95% confidential interval.

**Table 2:** Pharmacological characteristics of β_2_-adrenergic receptor agonists.

**β_2_-Adrenergic** **Receptor Agonists**	**EC_50_ (nM)**			
	**MCh**			
	**1 μM (*n* = 6)**		**10 μM (*n* = 8)**	
	**Mean ± S.D.**	**95% CI**	**Mean ± S.D.**	**95% CI**
Procaterol	1.9 ± 0.6	1.17–2.43	8.3 ± 2.6 *	6.13–10.47
Salbutamol	18.6 ± 6.3	11.69–25.21	296.4 ± 90.6 **	228.21–335.59
Formoterol	0.7 ± 0.3	0.39–1.02	2.1 ± 0.3 **	1.85–2.35
Isoproterenol	308.1 ± 68.6	236.08–380.12	1736.4 ± 98.2 **	1654.48–1818.52
**β_2_-Adrenergic** **Receptor Agonists**	**Emax (%)**			
	**MCh**			
	**1 μM (*n* = 6)**		**10 μM (*n* = 8)**	
	**Mean ± S.D.**	**95% CI**	**Mean ± S.D.**	**95% CI**
Procaterol	97.6 ± 2.2	95.40–99.81	70.2 ± 9.3 **	62.42–77.98
Salbutamol	78.6 ± 8.4	69.78–87.42	51.0 ± 6.4 **	45.65–56.35
Formoterol	98.1 ± 1.6	96.42–99.78	71.0 ± 8.1 **	64.23–77.77
Isoproterenol	100		100	

* *p* < 0.05, ** *p* < 0.01 vs. response to 1 μM MCh; Emax: maximal percent inhibition; 95% CI: 95% confidential interval.
